# Neoadjuvant systemic therapy for hepatocellular carcinoma

**DOI:** 10.3389/fimmu.2024.1355812

**Published:** 2024-03-01

**Authors:** R. Connor Chick, Samantha M. Ruff, Timothy M. Pawlik

**Affiliations:** Department of Surgery, Division of Surgical Oncology, The Ohio State University Wexner Medical Center and James Comprehensive Cancer Center, Columbus, OH, United States

**Keywords:** hepatocellular carcinoma, neoadjuvant, immunotherapy, targeted therapy, immune checkpoint inhibitor

## Abstract

Surgical resection and liver transplant remain the only curative therapies for most patients with hepatocellular carcinoma (HCC). Systemic therapy options have typically been ineffective, but recent advances, such as the combination of immune checkpoint inhibitors and targeted therapies, have shown great promise. Neoadjuvant systemic therapy in resectable or locally advanced HCC is under active investigation with encouraging results in small, early-phase trials. Many of these completed and ongoing trials include combinations of systemic therapy (e.g. immune checkpoint inhibitors, tyrosine kinase inhibitors), transarterial therapies, and radiation. Despite early successes, larger trials with evaluation of long-term oncologic outcomes are needed to determine the role of neoadjuvant systemic therapy in patients with HCC who may be eligible for curative intent surgery or transplant.

## Introduction

Hepatocellular carcinoma (HCC) is an aggressive primary liver tumor that most commonly arises in the setting of chronic liver inflammation or cirrhosis (1). Surgical resection or transplant remain the most effective treatments for patients with HCC. Patients who are not surgical candidates due to co-morbidities, liver dysfunction, or advanced disease rely on alternative options like locoregional therapies (e.g. transarterial therapies, percutaneous ablation) and/or systemic therapy. Additionally, many patients develop local or distant recurrence and will need systemic therapy (2). Sorafenib, a multi-targeted tyrosine kinase inhibitor (TKI), was the first effective systemic therapy for HCC and had remained the only systemic option for patients with advanced disease for over a decade until the REFLECT trial demonstrated noninferiority versus lenvatinib (3–5). Unfortunately, sorafenib and lenvatinib still only demonstrate an average survival of 12-14 months among patients with unresectable HCC. As such, there has been intense interest in identifying new systemic therapies to improve long term outcomes.

Immune checkpoint inhibitors (ICI) have proven to be effective in many solid tumors. Through interaction at immune checkpoints, cancer cells can inhibit T cells and evade the immune system, which can contribute to local progression or metastasis. ICIs block this interaction and restore the normal function of T cells (6, 7). The unique immune microenvironment of the liver plays a significant role in tumorigenesis and has been the focus of research efforts to evaluate the use of ICIs for HCC (8, 9). In the landmark IMbrave150 trial, patients with advanced HCC had improved survival with atezolizumab (PD-L1 inhibitor) and bevacizumab (vascular endothelial growth factor receptor (VEGFR) inhibitor) compared with sorafenib (10). This combination is now recommended as first-line therapy in the advanced setting (11). Additionally, the HIMALAYA trial demonstrated the efficacy of tremelimumab and durvalumab for patients with advanced HCC; in turn, this combination has also been approved as first-line therapy (12).

**Figure 1:**
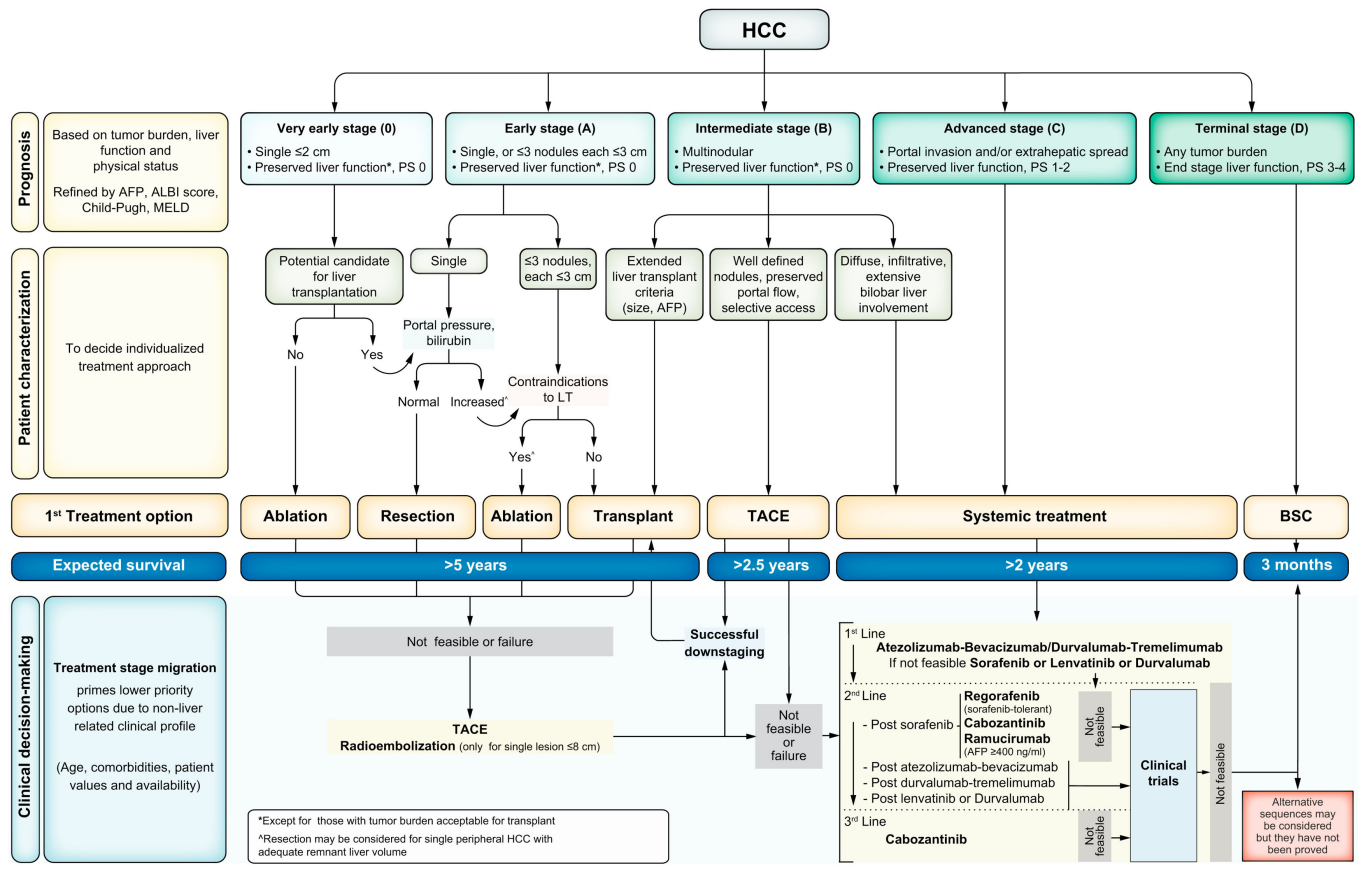
BCLC staging and first-line treatment algorithm. Liver function is evaluated using the Child–Pugh staging. AFP, alpha–fetoprotein; ALBI, albumin–bilirubin; BSC, best supportive care; ECOG-PS, Eastern Cooperative Oncology Group-performance status; LT, liver transplantation; MELD, model of end-stage liver disease; TACE, transarterial chemoembolization. This figure was reprinted with permission from reference 9. Appropriate copyright permission was obtained.

Given the success of ICI in the setting of advanced and metastatic HCC, studies are actively evaluating their use in the neoadjuvant and adjuvant setting. Currently, data on adjuvant or neoadjuvant options are relatively limited among patients with HCC (2). Recently, the combination of atezolizumab and bevacizumab demonstrated improved recurrence-free survival (RFS) in the adjuvant setting (13). In the neoadjuvant setting, ICI may allow for patients to be downstaged to be eligible for surgery or transplantation (14–17). Additionally, there is growing evidence that neoadjuvant immunotherapy may prime the immune system and prevent local or distant recurrence (18–23). The Barcelona Clinic Liver Cancer (BCLC) guidelines provide a logically designed, validated algorithm for initial management strategies across the spectrum of presentation of HCC (**Figure 1**) (11). Despite the recent success of systemic therapy combinations in advanced HCC, the optimal strategy for incorporating these therapies into the treatment paradigm for resectable and potentially resectable HCC has not been established. We herein review the published literature, as well as ongoing clinical trials focused on neoadjuvant systemic therapy for HCC.

## Neoadjuvant immunotherapy

### Tumor microenvironment in HCC

**Figure 2:**
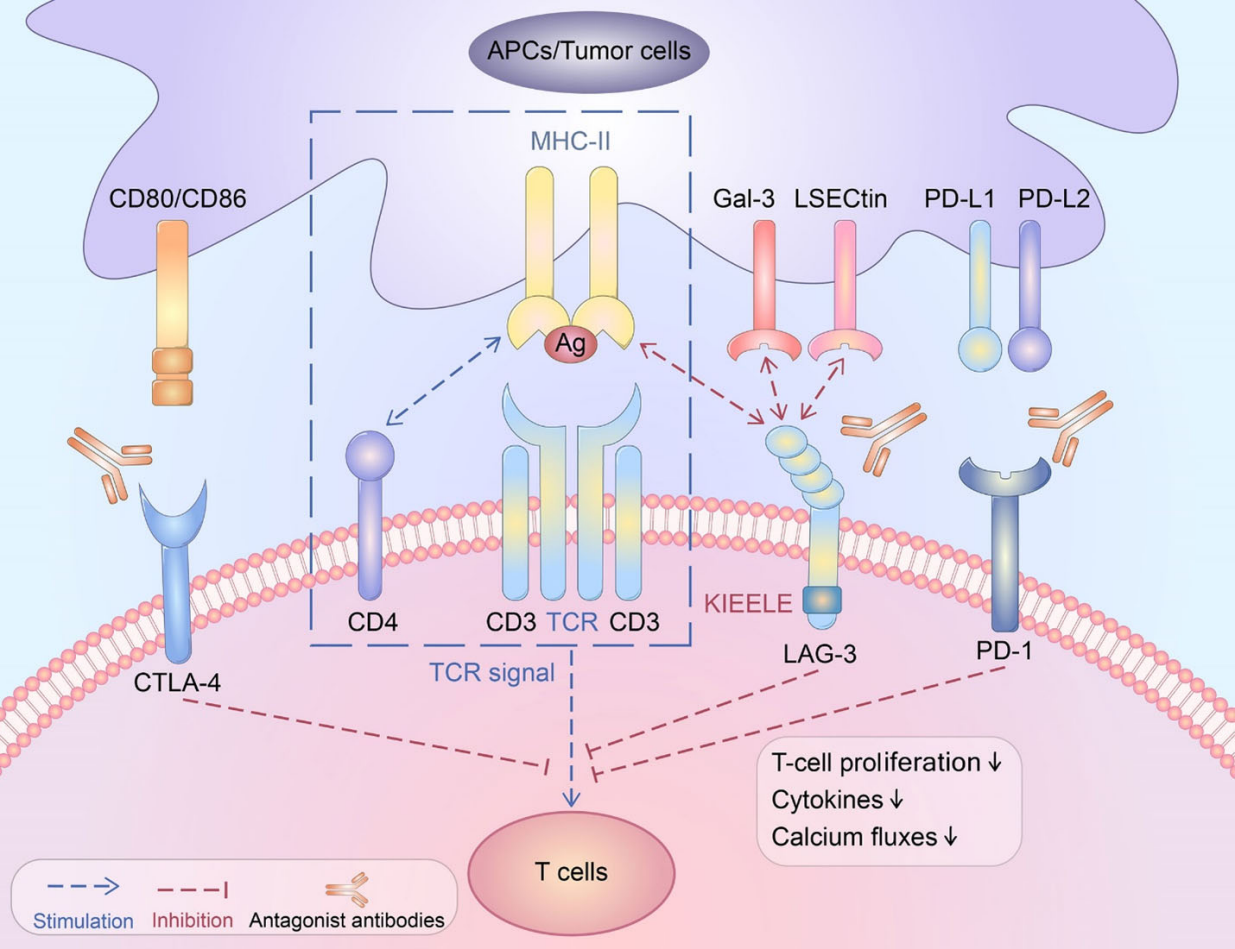
Schematic depicting various immune checkpoint ligands and corresponding receptors. CTLA-4, cytotoxic T lymphocyte antigen 4; CD, cluster of differentiation; TCR, T cell receptor; PD-1, programmed death receptor 1; MHC, major histocompatibility complex. This figure was reprinted from an open access publication (6).

The tumor microenvironment (TME) in hepato-pancreatico-biliary (HPB) cancers is a disordered inflammatory state characterized by infiltration of immune cells with paradoxically immunosuppressive phenotypes, such as regulatory T cells, exhausted CD4+ and CD8+ T cells, M2 macrophages, myeloid-derived suppressor cells (MDSC), and cancer-associated fibroblasts (CAF) (9). The T cells within the TME, referred to as tumor-infiltrating lymphocytes (TIL), are the primary target of immune checkpoint inhibitors (**Figure 2**). Many of these agents act on either the programmed death receptor 1 (PD-1) located on host T cells or the programmed death ligand 1 (PD-L1), which can be expressed by macrophages, some activated T cells, B cells, as well as certain tumor cells (24). Other ICI act on cytotoxic lymphocyte antigen 4 (CTLA-4), a receptor on host T cells that interacts with CD80/86 and inhibits MHC-II-dependent T cell receptor signaling (25). A third immune checkpoint that has been more recently described is LAG3, which is also a T cell surface receptor (26). Monoclonal antibodies targeting these checkpoints were initially used in immunogenic cancers such as melanoma, renal cell carcinoma, and non-small cell lung cancer, but are being widely studied in a variety of cancer types. These agents may be most applicable to cancers with PD-L1 expression, high tumor mutational burden, or DNA mismatch repair deficiency (27). However, the inherently tolerogenic microenvironment in the liver may represent another TME phenotype in which ICI may be effective (28). With established efficacy in the advanced setting, there is growing interest in the use of ICI in the neoadjuvant setting (29).

### Neoadjuvant immune checkpoint inhibition

Cemiplimab is a relatively newer monoclonal antibody against PD-1 and is currently used in advanced cutaneous squamous cell carcinoma (30). Marron et al. reported results from a single-arm, open-label, multicenter phase II study of neoadjuvant cemiplimab for patients with resectable HCC (31). This is part of a larger study of neoadjuvant cemiplimab for resectable HCC, non-small cell lung cancer (NSCLC), and head and neck squamous cell carcinoma (NCT03916627). This study enrolled 21 patients with resectable HCC between November 2019 and August 2020. Participants received two doses of preoperative cemiplimab, curative intent partial hepatectomy, and eight cycles of postoperative cemiplimab. The primary endpoint was significant tumor necrosis, defined as >70% necrosis on surgical pathology. Most patients (95%) underwent hepatectomy; one patient had surgery delayed due to pneumonitis. Among those individuals who underwent surgery, 4 (20%) had major pathologic response, defined as >70% necrosis, and three patients had 50-75% tumor necrosis (**Table 1**). Previous studies have demonstrated that the utility of RECIST 1.1 criteria is limited in patients on immunotherapy regimens (32). In line with these findings, this trial noted that on-treatment MRI was a better predictor of tumor necrosis than RECIST 1.1 criteria (32). This trial is currently ongoing and long term outcomes have not yet been reported.

**Table 1:** Existing data for neoadjuvant systemic therapy in HCC.

Lead Author	Year	Agent(s)	AE rate	Pathologic Response	Survival
Marron TU	2022	Cemiplimab	35% grade 3-4	20% MPR, 15% pCR	n/a
Kaseb AO	2022	Nivolumab +/- ipilimumab	43% grade 3-4 for combination, 23% grade 3-4 nivolumab alone	27% MPR for combination *vs*. 33% MPR nivolumab alone	Median PFS 19.5 *vs*. 9.4 months for combination *vs*. nivolumab alone
Nakajima M	2023	HPS70/GPC3 vaccine	20% grade 3-4	n/a	n/a
Guo C	2023	DEB-TACE + sintilimab	28% grade 3-4	49% MPR, 14% pCR	76% 1yr PFS
Ho WJ	2021	Nivolumab + cabozantinib	13.3% grade 3-4	42% MPR, 8.3% pCR	Median DFS 7.7 months for those with MPR
Xia Y	2022	Apatinib + camrelizumab	16.7% grade 3-4	17.6% MPR, 5.9% pCR	53.9% 1yr RFS

AE, adverse events; pCR, pathologic complete response; MPR, major pathologic response; PFS, progression-free survival; RFS, recurrence-free survival; DFS, disease-free survival.

There are three trials examining neoadjuvant single-agent ICI in HCC (**Table 2**). One (NCT05471674) has been completed; this study evaluated neoadjuvant nivolumab in borderline resectable HCC with a primary outcome of pathologic tumor response. Secondary endpoints include RFS, OS, short-term surgical outcomes, and safety. Results have not yet been reported. Two studies of neoadjuvant pembrolizumab in resectable HCC are ongoing, with primary outcomes including RFS and TIL from resected specimens.

**Table 2:** Neoadjuvant immunotherapy trials currently registered with clinicaltrials.gov.

NCT Number	Acronym	Study Status	Drug(s)	Resectability	Operative Goal
NCT05471674		Completed	Nivolumab	Potentially Resectable	Resection
NCT04224480		Recruiting	Pembrolizumab	Resectable	Resection
NCT03337841		Unknown	Pembrolizumab	Resectable	Resection
NCT05440864	NEOTOMA	Not Yet Recruiting	Durvalumab + Tremelimumab	Resectable	Resection
NCT03682276	PRIME-HCC		Ipilimumab + Nivolumab	Resectable	Resection
NCT03510871		Unknown	Ipilimumab + Nivolumab		Resection
NCT04658147		Recruiting	Nivolumab + Relatlimab (LAG3)	Potentially Resectable	Resection
NCT04123379		Active, Not Recruiting	Nivolumab + BMS-813160, BMS 986253 (CCR2/5)	Resectable	Resection

### Neoadjuvant combination checkpoint inhibition

Combining checkpoint inhibitors with complementary mechanisms of action has been well established in other disease sites. Kaseb et al. conducted an open-label phase II trial comparing perioperative nivolumab (anti-PD-1) alone to nivolumab and ipilimumab (anti-CTLA-4) in patients with resectable HCC (33). The primary endpoint was safety and tolerability, with secondary endpoints including response rate, as well as pathologic and immunologic correlates of response. Among 27 treated patients, 20 underwent surgical resection. Of note, none of the surgical cancellations were due to toxicity, but rather to disease progression (n=4), concern for insufficient future liver remnant (n=2), and prohibitive adhesive disease (n=1). As seen in trials of combination ipilimumab and nivolumab for other disease sites, immune-related adverse events were higher with combination therapy compared with monotherapy (34–36). Major pathologic response rate was 27% versus 33% for combination therapy versus monotherapy cohorts, respectively (33). However, long-term outcomes were improved with combination therapy, with median progression-free survival (PFS) 19.4 months for the combination therapy cohort versus 9.4 months for nivolumab alone (**Table 1**) (33).

The PRIME-HCC trial of preoperative nivolumab with ipilimumab is ongoing, but results from an interim analysis were presented at the American Society of Clinical Oncology (ASCO) annual meeting in 2022 (37). Among a safety report of 15 participants, one (7%) had a grade 3 or higher treatment-related adverse event. There were 13 patients with RECIST-evaluable preoperative imaging; objective response rate was 23%, with a disease control rate of 92%. Nine patients had evaluable pathology specimens, of which 78% had a pathologic response; pathologic complete response was seen in two (22%) (37). The full published results of this trial are eagerly anticipated, particularly in terms of long-term recurrence-free and overall survival (38).

Recently, a meta-analysis of neoadjuvant immune checkpoint inhibitors in HCC was published (39). This systematic review and meta-analysis included four published studies (31, 33, 40, 41), as well as five studies that were available only as conference abstracts (including PRIME-HCC described above) (37). In the pooled analysis, neoadjuvant ICI in HCC was beneficial in terms of pathologic response and recurrence-free survival, with decreased odds [odds ratio (OR) 0.17 (95% confidence interval (CI) 0.10-0.30)] of a pathologic complete response, and major pathologic response [OR 0.38 (95% CI 0.21-0.69)] in the absence of neoadjuvant ICI (39). The peer-reviewed published studies were weighted more heavily; however 34.6% of the weighted data related to pathologic complete response were based on data from conference abstracts only, which raises concerns about possible publication bias in the pooled results in the meta-analysis (39, 42). Many of the included studies also did not include a comparison group, which limited generalizability of the results in favor of neoadjuvant ICI (39).

In addition to PRIME-HCC, there are four other ongoing studies examining neoadjuvant combination immunotherapy for patients with HCC (**Table 2**). These studies include the NEOTOMA trial, which is evaluating tremelimumab and durvalumab in resectable HCC, and a different trial of a LAG3 inhibitor in addition to nivolumab among patients with potentially resectable HCC. Another trial combines nivolumab with a novel agent targeting the chemokines CCR2/CCR5, which may mitigate the effect of myeloid-derived suppressor cells (MDSC) in the TME (43). MDSC are immature host immune cells that tend to accumulate in the TME and suppress antitumor immunity through a variety of mechanisms (44). The prevalence of MDSC in the TME has been associated with poor outcomes among patients with HPB cancers (45). However, targeting MDSC by manipulating their chemotaxis can increase the immunogenicity of an otherwise immunologically cold TME (46). This unique immunotherapy combination is representative of a larger trend within cancer immunotherapy trials in which established agents such as checkpoint inhibitors are combined with other immune-modulating agents with distinct mechanisms.

### Neoadjuvant cancer vaccine

Cancer vaccines have been studied in solid tumors for many years, but have generally had low efficacy in most solid tumors, especially in the advanced setting (47, 48). Few trials have examined neoadjuvant vaccine strategies in HPB cancers with a few notable exceptions (49, 50). As monotherapy, these vaccines are generally well-tolerated, but have more efficacy when combined with ICI (51). Developed either as a personalized vaccine against the individual’s unique tumor-associated antigens (TAA) or with a prespecified TAA, vaccines are usually combined with an immune adjuvant. For example, among patients with HCC, heat shock protein 70 (HSP70) is a commonly utilized tumor antigen and the glypican-3 (GPC3) domain of carcinoembryonic antigen (CEA) has been proposed as a biomarker (52).

Nakajima et al. published results from the YCP02 trial, a phase I trial of a vaccine that combined HSP70, GPC3, a LAG3 antibody, and the immune adjuvant poly-ICLC. This vaccine was designed to elicit a T cell response to HSP70 and GPC3, using LAG3 checkpoint inhibition as a counter-regulatory mechanism; it was first demonstrated to be safe in a dose-escalation study among patients with metastatic gastrointestinal cancers (53). In a subsequent phase I trial, subjects with resectable HCC received 6 weekly vaccine doses preoperatively and 10 doses postoperatively (54). Vaccination was well tolerated (**Table 1**). Analysis of surgical pathology specimens demonstrated that 60% of participants (n=12) had infiltration of the tumor with CD8+ T cells specific for HSP70 and/or GPC3 (54). Further investigation is needed to determine whether this vaccine may prevent recurrence or prolong survival.

## Multimodality neoadjuvant therapy

There has been increased interest in combining different modalities with immune therapy, particularly in the neoadjuvant setting. Trans-arterial therapies, ICI, and TKI have been studied in the neoadjuvant setting with the goals of local downstaging and preventing recurrence. Combining ICI with other modalities may increase the efficacy of ICI by increasing immunogenicity in the neoadjuvant setting; this approach has been validated in preclinical studies in HPB cancers and in immunogenic solid tumors such as melanoma (20, 55, 56).

In a phase II trial, combination of immunotherapy with trans-arterial chemoembolization (TACE) was evaluated in the neoadjuvant setting for patients with HCC (57). Drug-eluting bead TACE (DEB-TACE) was combined with sintilimab, a PD-1 inhibitor, with the rationale that TACE will cause the release of neoantigens and subsequently make HCC more immunogenic. In turn, this process may increase the efficacy of ICI therapy. Sixty patients with BCLC A or B outside the Milan criteria were treated with this combination. The majority of participants were male (85%) and had cirrhosis secondary to hepatitis B infection (84%). At a median follow-up of 26 months, median PFS was 30.5 months, with a 12-month PFS estimate of 75%. Among all treated patients, objective response rate was 62%. Ultimately 51 patients proceeded to surgical resection and 7 (14%) had a pathologic complete response (**Table 1**). These patients also had a corresponding decrease in tumor markers, including AFP and PIVKA-II (57).

The combination of ICI with tyrosine kinase inhibitors has gained increasing interest in the neoadjuvant setting in HCC. Nivolumab with cabozantinib, a multi-kinase inhibitor with activity against VEGFR2, was studied in the neoadjuvant setting with the goal of converting unresectable HCC to resectable and increasing antitumor immunity (40). In this single-center phase 1b trial, 15 patients with borderline resectable or locally advanced HCC were given 8 weeks of neoadjuvant therapy. Surgery was attempted in 13 of 15 patients and completed in 12; 5 patients had major pathologic response (**Table 1**). This study met its primary endpoint by demonstrating feasibility of this neoadjuvant therapy regimen (40).

Xia et al. reported a single-arm, open-label, phase II trial evaluating camrelizumab (anti-PD-1) and apatinib (a VEGFR2 inhibitor) in the preoperative setting for patients with resectable HCC (41). This combination was well tolerated among the 18 enrolled participants, with only 16% experiencing a grade 3 or 4 adverse event. Major pathologic response rate, defined as >90% tumor necrosis, occurred in 17.6% of participants. RFS at one year was 53.9% (**Table 1**). Exploratory analyses of circulating tumor DNA (ctDNA) suggested a correlation between decrease in ctDNA levels and response to neoadjuvant therapy, whereas rising ctDNA during adjuvant therapy was associated with recurrence (41).

There are many ongoing trials involving combination NAT (**Table 3**). Of the 34 trials identified, 28 (82.3%) involve at least one immune checkpoint inhibitor, and 26 (76.5%) involve at least one tyrosine kinase inhibitor. There were 10 studies (29.4%) utilizing three or more modalities, including ICI, TKI, transarterial therapies, hepatic arterial infusion chemotherapy (HAIC), systemic chemotherapy, and SBRT. The majority of these studies (n=44, 88%) give NAT prior to curative intent resection, with the remainder (n=6, 12%) prior to transplant.

**Table 3:** Combination neoadjuvant therapy trials currently registered with clinicaltrials.gov.

NCT Number	Acronym	Study Status	Modalities	Drug(s)	Resectability	Operative Goal
NCT04954339	DYNAMIC	Recruiting	TKI + ICI	Atezolizumab + Bevacizumab	Potentially Resectable	Resection
NCT03299946		Completed	TKI + ICI	Cabozantinib + Nivolumab	Potentially Resectable	Resection
NCT04888546		Recruiting	TKI + ICI	Anlotinib + TQB2450 (PD-L1)	Resectable	Resection
NCT04521153		Recruiting	TKI + ICI	Apatinib + Camrelizumab	Resectable	Resection
NCT04930315	CAPT	Recruiting	TKI + ICI	Apatinib + SHR-1210 (PD-1)	Resectable	Resection
NCT04297202	HCC-009	Unknown	TKI + ICI	Apatinib + SHR-1210 (PD-1)	Resectable	Resection
NCT05908786		Recruiting	TKI + ICI	Atezo + Bev + Tiragolumab (TIGIT(ICI)) + Tobemstomig (PD-L1xLAG3 bispecific)	Resectable	Resection
NCT05389527	NeoLeap-HCC	Active, Not Recruiting	TKI + ICI	Lenvatinib + Pembrolizumab	Resectable	Resection
NCT05807776		Not Yet Recruiting	TKI + ICI	Lenvatinib + Tislelizumab	Resectable	Resection
NCT04615143	TALENT	Recruiting	TKI + ICI	Lenvatinib + Tislelizumab	Resectable	Resection
NCT03867370		Unknown	TKI + ICI	Lenvatinib + Toripalimab	Resectable	Resection
NCT05185505		Recruiting	TKI + ICI	Atezolizumab + Bevacizumab		Transplant
NCT04443322	Dulect2020-1	Recruiting	TKI + ICI	Lenvatinib + Durvalumab		Transplant
NCT04425226	PLENTY202001	Recruiting	TKI + ICI	Lenvatinib + Pembrolizumab		Transplant
NCT04850040		Not Yet Recruiting	TKI + ICI + Chemo	Apatinib + Camrelizumab + Oxaliplatin	Potentially Resectable	Resection
NCT05339581	iPLENTY-pvtt	Not Yet Recruiting	TKI + ICI + IMRT	Lenvatinib + Pembrolizumab, Sintilimab, Camrelizumab, or Tislelizumab	Potentially Resectable	Transplant
NCT04857684		Recruiting	TKI + ICI + SBRT	Atezolizumab + Bevacizumab	Resectable	Resection
NCT05613478		Recruiting	TKI + ICI + TAE	Apatinib + Camrelizumab	Resectable	Resection
NCT05250843		Recruiting	TKI + ICI + TAE	Lenvatinib + Sentilimab	Resectable	Resection
NCT05920863		Recruiting	TKI + ICI + TAE	Lenvatinib + Tislelizumab	Resectable	Resection
NCT06003673		Recruiting	TKI + ICI + TAE	Lenvatinib + Tislelizumab	Resectable	Resection
NCT05225116		Recruiting	TKI + ICI + XRT	Lenvatinib + Sintilimab	Resectable	Resection
NCT05621499		Not Yet Recruiting	TKI + ICI or HAIC	Lenvatinib + Sintilimab	Resectable	Resection
NCT05137899	ADVANCE HCC	Recruiting	TKI + ICI *vs* XRT	Atezolizumab + Bevacizumab	Potentially Resectable	Resection

## Discussion and future directions

Neoadjuvant systemic therapy in HCC has demonstrated early success in clinical trials. These early phase neoadjuvant trials have laid the groundwork for further expansion in future clinical trials focused on combination neoadjuvant therapy. Future efforts will likely aim to incorporate targeted therapies, immunotherapies, and locoregional therapies to prime the immune system and downstage locally advanced tumors.

Preclinical and correlative studies bolster the theory that NAT will lead to a local tumor response and prevent recurrence by altering the TME; this process has been observed both with immune therapies and other agents (58). The TME of HCC warrants special consideration given the liver’s complex immune system. In a healthy liver, the immune microenvironment is tightly regulated due to constant exposure to both self and non-self antigens (e.g. dietary and bacterial products) (28). In the setting of chronic inflammation, the microenvironment is altered and reflects a state of immune cell exhaustion. This process makes the liver more susceptible to the growth and expansion of malignant cells. As such, HCC commonly arises in patients with cirrhosis, which is due to chronic inflammation from various etiologies (e.g. alcohol or fat consumption, chronic hepatitis) (59). When cancer cells evade the immune system through manipulation of immune checkpoints, the underlying problem can be exacerbated. As such, ICIs can disrupt the HCC TME to restore immune cell function against cancer cells (28).

Several synergistic mechanisms for the efficacy of neoadjuvant immunotherapy in HCC have been described. One consistent finding with these trials and in other disease sites is the formation of tertiary lymphoid structures (TLS), which serve as the locus for generating T cell memory and are associated with improved survival (60–63). Importantly, examination of TLS was one of the correlative analyses in the aforementioned study of cabozantinib and nivolumab, in which responders had a higher density of TLS as well as more tumor-specific CD4+ and CD8+ T cells (40). A further analysis of these patients was conducted using spatial transcriptomics, which identified cancer-associated fibroblasts, intratumor inflammatory markers, and modulation of the extracellular matrix as predictors of response (58). Additionally, the co-localization of PAX5, a regulator of B cell maturation, with areas of increased B cell gene expression suggests an important role for these B cells in response to immunotherapy (58). Knowledge of the role of B cells in the tumor microenvironment is evolving, as most of the focus on the role of TIL in the TME has been on tumor infiltrating T lymphocytes. These so-called TIL-Bs play an important role in supporting the development of tumor-specific T cells as well as promoting innate antitumor immunity by recruiting and reprogramming natural killer cells and macrophages (64).

These immunologic correlates of response to ICI are well described in the setting of surgical resection, yet there are unanswered questions about the feasibility of ICI prior to transplant. For example, it has yet to be established whether preoperative immunotherapy is compatible with postoperative immunosuppression, and whether this approach affects oncologic outcomes, risk of rejection, or immunosuppression-related infections or cancers (65).

While there are many future directions for studying neoadjuvant systemic therapy in HCC, perhaps the two most exciting opportunities involve priming the immune system to prevent recurrence and downstaging locally advanced tumors to allow for resection or transplant. While the cabozantinib and nivolumab study examined both topics, it was a small phase 1b trial and larger trials are needed to confirm these results. There are other studies of combination therapies that have enrolled patients with advanced disease, such as portal vein invasion or individuals otherwise ineligible for resection or transplant; these studies have reported impressive results with downstaging. For example, a phase II trial of lenvatinib and toripalimab with hepatic artery infusion chemotherapy with 5-fluorouracil and oxaliplatin (FOLFOX) demonstrated an ORR of 66.7%; perhaps more interestingly, 22.2% of participants became eligible for resection or transplant despite having locally advanced disease at presentation (66).

Adaptive trial design may be a more efficient strategy to evaluate whether neoadjuvant systemic therapy with or without local therapies may be able to downstage locally advanced HCC to allow for resection or transplant. Eligibility for resection or transplant, response to neoadjuvant therapy, and/or relevant biomarkers may be able to stratify participants into different treatment arms within the same trial. An example of adaptive trial design in neoadjuvant therapy is the OPRA trial in rectal cancer, where participants were assigned to a surgical treatment arm only after post-NAT restaging (67). Another example is in pancreatic cancer, where the effect of NAT on resectability is a crucial and dynamic component of treatment selection and sequencing; therefore neoadjuvant trials such as SWOG S-1505 are designed in a way to allow reassessment of resectability after NAT (16, 23, 68). In HCC, determination of resectability may be improved by dynamic assessment over time. Thus, patients with locally advanced HCC may be a population in which NAT will become the most useful. To further this research, trials must thoroughly investigate biomarkers including immunologic correlates and ctDNA in addition to long term survival outcomes. In turn, this approach will allow for better selection of patient populations who will benefit the most from NAT.

There may be safety considerations with implementing NAT prior to major liver resection or transplant. In particular, many of these combinations contain TKIs; those agents with the most efficacy in HCC either preferentially (sorafenib, lenvatinib) or selectively (apatinib) inhibit angiogenesis and related growth factor signaling. Interestingly, these agents seem to have a safer perioperative safety profile than bevacizumab, which has been shown to increase the risk of post-operative morbidity and mortality (69, 70). The effect of ICI alone on surgical outcomes is less clear. The PRIME-HCC trial using both nivolumab and ipilimumab will provide interesting insight from this standpoint (38). While it is unlikely that surgical complications would increase as a direct result of ICI, immune-related adverse events such as endocrinopathies, pneumonitis, or even hepatitis may have important implications for perioperative outcomes (71).

Some limitations common to the currently available evidence for NAT in HCC include small sample size, heterogeneous inclusion criteria, and non-uniform outcome measures, such as varying definitions of “major pathologic response.” Ultimately, large, multi-center trials with long-term oncologic outcomes are needed to demonstrate the utility of neoadjuvant systemic therapy in patients with HCC. These trials should evaluate long-term outcomes such as overall survival and RFS, surrogate markers such as pathologic response and local downstaging, and immunologic and tumor-intrinsic biomarkers.

## Author contributions

RC: Writing – original draft. SR: Writing – review & editing. TP: Writing – review & editing.
